# La tumeur adénomatoïde odontogène: à propos de deux observations

**DOI:** 10.11604/pamj.2021.38.386.22898

**Published:** 2021-04-20

**Authors:** Ahlem Bchir, Ahlem Bdioui, Sarra Mestiri, Samia Ayachi, Hbib Khochtali, Sihem Hmissa, Moncef Mokni

**Affiliations:** 1 Laboratoire d’Anatomie et Cytologie Pathologique, Centre Hospitalier Universitaire Farhat Hached, Sousse, Tunisie,; 2 Laboratoire d´Anatomie et Cytologie Pathologique, Centre Hospitalier Universitaire Sahloul, Sousse, Tunisie,; 3 Service de Chirurgie Maxillo-Faciale, Centre Hospitalier Universitaire Sahloul, Sousse, Tunisie

**Keywords:** Tumeur adenomatoïde odontogène, bénigne, tumeurs odontogéniques, rapport de cas, Adenomatoid odontogenic tumor, benign, odontogenic tumors, case report

## Abstract

La tumeur adénomatoïde odontogène est une tumeur épithéliale bénigne, qui touche essentiellement la femme jeune, elle siège habituellement dans la partie antérieure du maxillaire supérieur. Son diagnostic peut être suspecté cliniquement devant une formation kystique, associée à une dent incluse, mais la confirmation repose sur l'examen histopathologie. Il s'agit de deux patients précédemment de 13 et de 37 ans, sans antécédents, qui consultaient suite à une tuméfaction siégeant respectivement au niveau du maxillaire et au niveau de la mandibule. L'examen anatomo-pathologique de ces lésions avait conclu à une tumeur adénomatoïde odontogène. A travers ces deux observations, nous rapportons les caractéristiques anatomo-cliniques, l'évolution et le traitement de ce type de tumeur.

## Abstract (en)

Adenomatoid odontogenic tumor is a benign epithelial tumor which mainly affects young women. It usually occurs in the anterior portion of the upper jaw. Diagnosis can be clinically suspected in patients with cyst formation associated with retained tooth but histopathological confirmation is required. This study involved two patients aged 13 and 37 years, with no previous history, presenting after the occurrence of a swelling on the jaw and mandible. Anatomopathological examination of these lesions showed adenomatoid odontogenic tumor. This study highlights the anatomoclinical features, outcome and treatment of this type of tumor.

## Introduction

La tumeur adénomatoïde odontogène est une tumeur épithéliale bénigne rare, qui touche essentiellement la femme jeune. Elle siège habituellement dans la partie antérieure du maxillaire supérieur [1]. Son diagnostic peut être suspecté cliniquement devant une formation kystique évoluant non douloureuse et lente associée à une dent incluse [2, 3], mais la confirmation repose sur l´examen histologique. Le traitement consiste en une simple énucléation [2].

## Patient et observation

Nous rapportons deux nouveaux cas de tumeur adénomatoïde odontogène diagnostiqués au Service d´Anatomie et de Cytologie Pathologiques de l´Hôpital Farhat Hached de Sousse.

### 1^e^ observation

**Figure 1:**
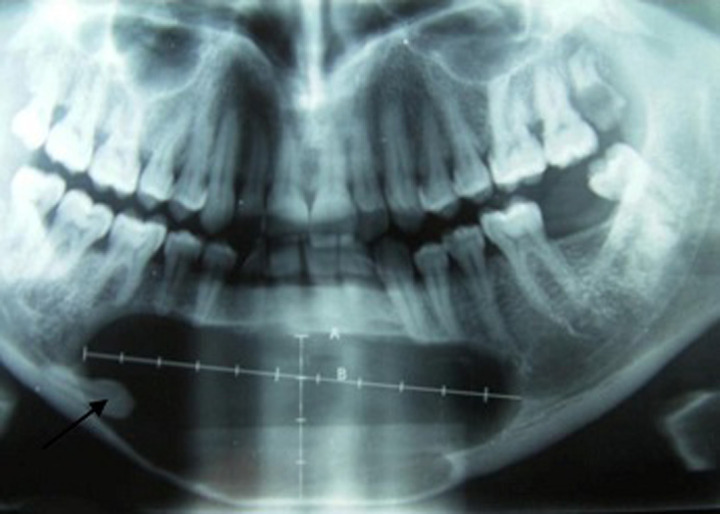
radiographie panoramique volumineuse lésion kystique ostéolytique, s’étendant de la 35^e^ à la 46^e^ avec amincissement de la corticale osseuse en regard et présence d’une canine incluse (flèche)

**Figure 2:**
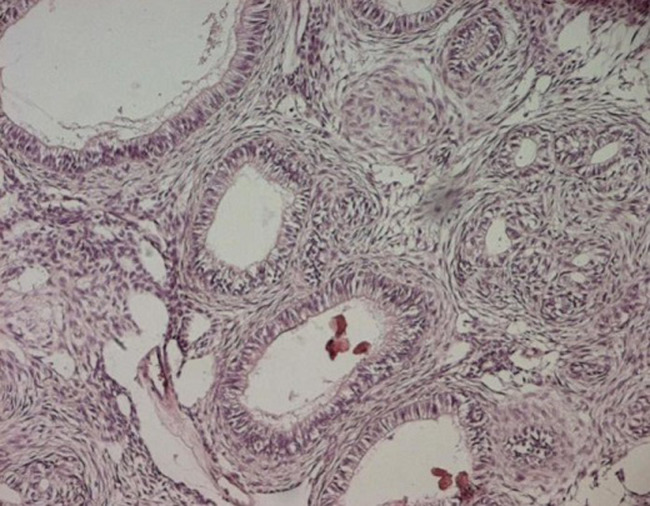
H.E. x 250 prolifération tumorale bien circonscrite, organisée en lobules, en amas et en glandes, parfois centrées par un matériel éosinophile ou par des calcifications (flèche)

**Figure 3:**
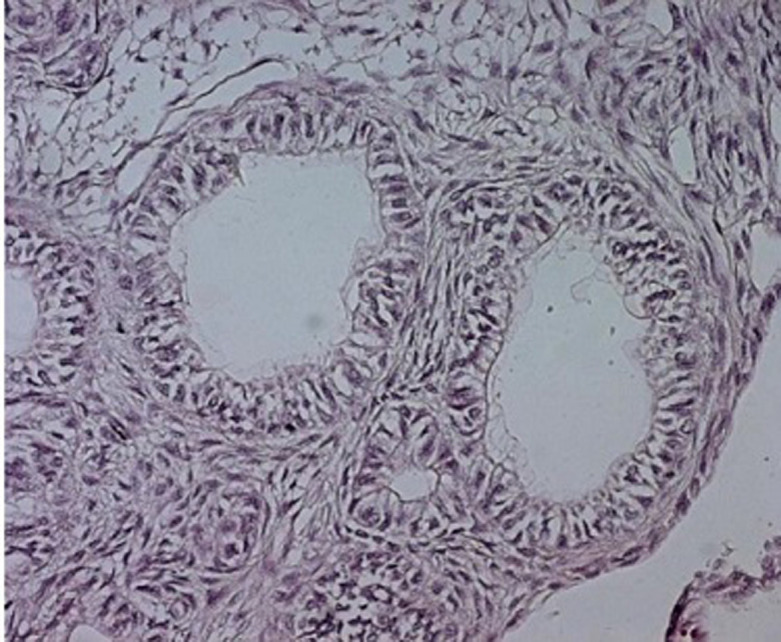
H.E. x 400 les cellules tumorales sont cylindriques, à cytoplasme abondant clair et à noyau régulier, polarisé, pourvu d’une chromatine fine; le stroma est cellulaire, fait de cellules fusiformes, régulières

Patient âgé de 37 ans, sans antécédents pathologiques notables, ayant consulté à la suite de l´apparition d´une tuméfaction mandibulaire symphysaire, évoluant lentement depuis plusieurs années. A l´examen exo-buccal, il existait une tuméfaction mandibulaire symphysaire qui s´étend aux 2 branches horizontales et mesure 5 x 6cm. La peau en regard était saine. La sensibilité labio-mentonnière était conservée. A l´examen endo-buccal, la tuméfaction comblait le vestibule. Elle était de consistance rénitente, indolore à la palpation. La muqueuse en regard était saine et il n´y avait pas de mobilité dentaire associée. Les aires ganglionnaires cervicales et sub-mandibulaires étaient libres. Le reste de l´examen physique était normal. La radiographie panoramique montrait une volumineuse lésion kystique ostéolytique, s´étendant de la 35^e^ prémolaire à 46^e^ molaire avec amincissement de la corticale osseuse, une rhysolyse dentaire et une dent incluse (la 43^e^ canine) (Figure 1). A l´examen tomodensitométrie (TDM), on retrouvait un volumineux kyste mandibulaire, mesurant 10,8 x 3,8cm, soufflant la corticale et l´interrompant par places (Figure 2). Cet aspect évoquait un kyste folliculaire ou un kératokyste. Le patient a eu une énucléation avec extraction de la dent incluse. A l´examen macroscopique, on a reçu une formation kystique, mesurant 6cm de diamètre, à paroi grisâtre, à surface externe lisse, uniloculaire et comportant des végétations dont la taille variait de 0.2 à 1cm. L´examen histologique a conclu à une tumeur adénomatoïde odontogène, devant la présence d´une prolifération tumorale bien circonscrite, d´aspect biphasique (Figure 3). La première composante était organisée en lobules, en amas et en glandes, parfois centrées par un matériel éosinophile. Les cellules tumorales étaient cubiques ou cylindriques, à cytoplasme abondant clair et à noyau régulier, polarisé, pourvu d´une chromatine fine. La deuxième composante correspond à un stroma cellulaire, fait de cellules fusiformes, dépourvues d´atypies. Il y avait de nombreuses calcifications. Après un recul de 18 mois, l´évolution était favorable.

### 2^e^ observation

Adolescent âgé de 13 ans, sans antécédents particuliers, qui présentait depuis un an une tuméfaction palatine isolée. A l´examen exo-buccal, il existait une tuméfaction soulevant le seuil narinaire gauche et effaçant le sillon naso-génien gauche. L´examen endo-buccal objectivait une tuméfaction de 3cm de grand axe, à double versant vestibulaire et palatin, étendue de la 21^e^ incisive à la 24^e^ prémolaire. Elle était dure, fixe, indolore avec une muqueuse d´aspect normal en regard. La 22^e^ incisive était absente. Le scanner du massif facial objectivait une lésion ostéolytique maxillaire gauche arrondie de 1,5cm de diamètre, de densité liquidienne, a contours nets et réguliers. Elle était centrée par une dent incluse (la 22^e^), qui était déjetée en avant et entourée par de fines calcifications, disposées en couronne. La lésion soufflait la corticale, qui venait au contact du cornet inferieur gauche et respectait le sinus maxillaire. Cet aspect radiologique évoquait un kyste péri-coronaire. Le patient a eu une énucléation du kyste. A la macroscopie, on a reçu une canine de 1.5cm à laquelle était appendue une formation kystique de 2.3cm, à contenu charnu et friable, la paroi était épaisse, grisâtre et d´aspect luisant. L´examen histologique a conclu à une tumeur adénomatoïde odontogène, de morphologie similaire à celle décrite précédemment. Après un recul de 6 mois, l´évolution était favorable.

## Discussion

La tumeur adénomatoide odontogène est une tumeur bénigne rare, classée parmi les tumeurs odontogéniques épithéliales et conjonctives avec ou sans formation de tissu dentaire dur [3-7]. Elle représente environ 3 à 7% de l´ensemble des tumeurs odontogéniques [2-4, 8, 9]. L´âge de découverte varie entre 10 et 20 ans [2-4, 6, 8]. Le sexe-ratio M/F varie de 1/2 à 1/9 [2-4, 6, 8, 9]. L´atteinte du maxillaire est plus fréquente que la localisation mandibulaire [3, 4, 6, 8], avec une prédilection pour la partie antérieure [3, 4, 9]. On distingue deux formes [3]; la forme centrale ou centro-osseuse: c´est la forme commune (97% des cas), associée dans 73% des cas à une dent incluse; la deuxième forme est dite périphérique ou extra-osseuse: c´est une forme rare, qui se traduit cliniquement par une tumeur gingivale ressemblant à une épulis fibreuse.

La pathogénie est encore mal connue, mais la ressemblance entre les cellules cylindriques de la composante épithéliale et les améloblastes, ainsi que l´association fréquente avec les dents incluses, poussent à croire que la tumeur proviendrait de l´épithélium dentaire. Cette notion expliquerait la confusion radio-clinique entre la tumeur adénomatoïde odontogène et le kyste folliculaire [4, 8]. Cliniquement, au stade de début, la découverte est souvent fortuite lors d´un examen radiographique de routine ou réalisé à la suite d´un retard d´éruption dentaire ou d´un diastème inter dentaire [7]. Quand la lésion devient symptomatique, elle se manifeste par une tuméfaction osseuse indolore, de consistance ferme, augmentant lentement de volume [4, 9]. L´aspect radiologique de la tumeur adénomatoïde odontogène est variable. Dans sa forme centrale, elle apparaît comme une image uniloculaire radio-transparente, bien circonscrite par une paroi radio-opaque associée à une dent incluse, le plus souvent la canine maxillaire, rappelant un kyste dentigère.

La forme périphérique se présente comme une lésion radio-transparente se trouvant au contact des racines dentaires. En fonction de la localisation, elle peut simuler un kyste résiduel, un kyste globulo-maxillaire, un kyste parodontal latéral, un kératokyste ou même un améloblastome. Lorsqu´il existe des calcifications, le diagnostic différentiel radiologique fait évoquer un kyste odontogène calcifié ou une tumeur épithéliale odontogène calcifiée [2, 3]. Macroscopiquement, la taille varie entre 2 et 9cm [3]. La tumeur est bien circonscrite par une capsule fibreuse conjonctive, ayant une surface lisse et une consistance assez ferme. Une dent peut l´être attachée. A la coupe, il est fréquent de retrouver dans la lumière du kyste un liquide séro-hématique avec des végétations [4]. Seul l´histologie permet de confirmer le diagnostic [3]. Histologiquement, la tumeur odontogène adénomatoïde est caractérisée par des lobules, des travées, creusées de cavités pseudo-glandulaires dont la lumière renferme un matériel éosinophile amorphe. Les cellules épithéliales sont cylindriques, munies de noyaux polarisés, situés au contact du pole apical. Le stroma est peu abondant, grêle, fibreux [2, 4, 5, 9]. L´étude immunohistochimique n´a pas d´intérêt [5]. L´association de la tumeur adénomatoide odontogène à d´autres tumeurs odontogènes a été observée, notamment avec la tumeur épithéliale odontogène calcifiée, le kyste odontogène calcifié, le kyste folliculaire et l´améloblastome [3]. Le traitement repose sur l´énucléation. Aucune récidive n´a été signalée [3, 4].

## Conclusion

La tumeur adénomatoide odontogène est une lésion constamment bénigne, d´évolution lente. La présentation clinique et l´aspect radiologique non caractéristiques sont la source d´une difficulté d´orientation diagnostique préopératoire. Elle doit être suspectée devant un tableau de kyste folliculaire ou bien des images radio-transparentes au niveau des maxillaires supérieurs. Particulièrement, si elle est associée à une dent incluse. Le diagnostic de certitude repose uniquement sur l´examen histologique.
